# Machine learning-based identification of high-risk triple-negative breast cancer patients despite pathological complete response after neoadjuvant chemotherapy

**DOI:** 10.1038/s41523-026-00979-0

**Published:** 2026-06-02

**Authors:** Koen Kwakkenbos, Nadine S. van den Ende, Bernadette A. M. Heemskerk-Gerritsen, Jifke F. Veenland, Agnes Jager, Carolien H. M. van Deurzen

**Affiliations:** 1 Department of Pathology and Clinical Bioinformatics, Erasmus MC Cancer Institute, Erasmus University Medical Centre, Rotterdam, The Netherlands; 2 Department of Medical Oncology, Erasmus MC Cancer Institute, Erasmus University Medical Centre, Rotterdam, The Netherlands; 3 Department of Radiology and Nuclear Medicine, Erasmus University Medical Centre, Rotterdam, The Netherlands

**Keywords:** Biomarkers, Cancer, Oncology

## Abstract

The binary pathological complete response (pCR) status of triple-negative breast cancer (TNBC) patients after neoadjuvant chemotherapy (NAC) inadequately captures risk heterogeneity for prognosis. Therefore, this study aimed to develop a machine learning-based survival model to predict individualized overall survival (OS) in TNBC patients with pCR after NAC. This retrospective study utilized a Dutch nationwide cohort of TNBC patients, diagnosed between 2013 and 2021. We trained a survival model on the data using nested 5-fold cross-validation. Patients were stratified into a low- and high-risk group, based on the predicted survival probabilities. A total of 2642 patients with a pCR post-NAC were included, of whom 2620 were used in the final analysis. The survival model achieved good discrimination (C-index 0.754). Risk stratification identified a high-risk subgroup (*n* = 495, 18.9%) with significantly worse survival compared to the low-risk group (5-year OS 85.2% vs. 96.7%, log-rank *P* < 0.001). Pre-treatment nodal status (cN) was the strongest predictor, followed by age and clinical tumor stage (cT). This nationwide analysis demonstrates that TNBC patients achieving pCR remain a heterogeneous group with distinct survival outcomes. Machine learning-based survival modeling may enable individualized risk prediction using routinely collected clinical variables and may guide future post-surgical treatment escalation for high-risk TNBC patients despite pCR.

## Introduction

Breast cancer remains the leading cause of cancer mortality for women worldwide, with approximately 15% of the cases classified as triple-negative breast cancer (TNBC)^1^. This subset is defined by the lack of the estrogen receptor (ER) and progesterone receptor (PR), as well as the absence of human epidermal growth factor receptor 2 (HER2) overexpression^2^. TNBC is an aggressive subtype, characterized by higher recurrence rates and risk of death compared to other breast cancer subtypes^2^. In contrast to hormone receptor-positive or HER2-positive disease, TNBC has no established targeted adjuvant treatments, which makes patients more reliant on the response to neoadjuvant chemotherapy (NAC) for long-term prognosis. Patients are increasingly treated with NAC^3^, possibly combined with immunotherapy dependent on tumor stage^4,5^.

A principal goal of NAC is to achieve a pathological complete response (pCR), which is the strongest surrogate marker for favorable outcomes after TNBC^6,7^. However, despite this positive association, around 10% of patients who achieve pCR will still experience disease relapse^8,9^. Currently, effective adjuvant treatment options exist for TNBC patients with residual disease (RD) after NAC^10,11^. However, pCR patients with a high risk of relapse may also benefit from adjuvant therapy escalation. Current risk stratification, based solely on the distinction between pCR versus RD, fails to identify these individuals with pCR at risk of relapse. It is known that the recurrence-risk also depends on pre-NAC tumor characteristics and choice of therapy^12,13^. Therefore, identifying high-risk patients among those achieving pCR requires prognostic models that also use information from the pre-NAC situation.

Existing predictive models like CancerMath and PREDICT currently do not integrate the changes in clinical (pre-NAC) and pathological tumor characteristics post-NAC^14,15^. This limitation underscores the need for more advanced models that integrate pre- and post-NAC data to provide more accurate and personalized risk assessments. Advanced survival analysis using machine learning offers an approach to learn complex relationships between pre- and post-NAC variables to generate an individualized risk score.

The aim of this current study is to develop an advanced machine learning-based survival model that takes pre-NAC data into account for patients who achieved pCR. This model could be used to potentially identify pCR patients who might benefit from post-surgery treatment escalation.

## Results

### Patient and tumor characteristics

The median age at diagnosis was 49 years (Table 1). The majority (91.7%) of patients presented with a cT1 or cT2 tumor, and most were clinically node-negative (cN0, 67.3%). Nearly all patients received combination anthracycline and taxane-based NAC (94.2%) with a median treatment duration of 19 weeks. Breast-conserving surgery was performed in 68.4% of patients, while 31.6% underwent mastectomy. The predominant histological subtype was invasive carcinoma of no special type (NST, 94.4%), and most tumors were grade 3 (80.7%).

**Table 1:** Characteristics of the complete cohort

Variable	Number of patients (*n* = 2642)
**Number of deaths (events) [*n*** **(%)]**	131 (5.0)
**Median follow-up time [years]**	4.4
**Age [Median (IQR)]**	49.0 (41.0–57.0)
**cT stage [*n*** **(%)]**	
1	747 (28.3)
2	1674 (63.4)
3	155 (5.9)
4	64 (2.4)
*Missing*	*2*
**cN stage [*n*** **(%)]**	
0	1769 (67.3)
1	611 (23.3)
2	61 (2.3)
3	186 (7.1)
*Missing*	*15*
**Tumor multifocality, present [*n*** **(%)]**	402 (15.2)
**Tumor histological subtype [*n*** **(%)]**	
NST (no special type)	2479 (94.4)
Special subtypes	76 (5.6)
*Missing*	*16*
**Tumor grade [*n*** **(%)]**	
I	13 (0.6)
II	385 (18.7)
III	1663 (80.7)
*Missing*	*581*

*pCR* pathological complete response, *cT* clinical tumor stage, *cN* clinical node stage, *NAC* neoadjuvant chemotherapy, *IQR* interquartile range.

### Survival model development and performance

The CoxNetSurvivalAnalysis model showed the best performance for predicting OS. This model achieved a mean C-index of 0.754 (SD: 0.015). The time-dependent AUC was 0.83 (SD: 0.06) at 1 year, 0.76 (SD: 0.02) at 3 years, and 0.75 (SD: 0.03) at 5 years post-surgery (Supplementary Fig. 1). The calibration, as measured through the IBS, was 0.033 (SD: 0.001). The most important predictor of survival was the cN stage (0.111, SD: 0.018), followed by age at breast cancer diagnosis, cT stage, and duration of NAC, although these had substantially smaller impacts on the model’s performance (0.023 (SD: 0.014); 0.019 (SD: 0.010); and 0.014 (SD: 0.007), respectively; Table 2). Other clinicopathological variables, specifically tumor histological subtype and tumor grade, were evaluated but did not improve the model’s predictive performance. This is likely attributable to the high prevalence of the NST subtype (94.4%) and the high proportion of high-grade tumors (80.7%).

**Table 2:** Univariate and multivariate hazard ratio’s (HR) and corresponding 95% confidence interval (95% CI) of the included variables calculated on the full cohort

Variable	Univariate HR (95% CI)	Univariate *P*-value	Multivariate HR (95% CI)	Multivariate *P*-value	Impact on C-index (SD)
cN stage	2.01 (1.75–2.31)	<0.001	1.73 (1.49–2.02)	<0.001	0.111 (0.018)
Age	1.03 (1.01–1.04)	<0.001	1.03 (1.01–1.04)	0.001	0.023 (0.014)
cT stage	2.13 (1.72–2.64)	<0.001	1.45 (1.11–1.81)	0.001	0.019 (0.010)
Duration of NAC	1.04 (0.99–1.09	0.088	1.04 (0.99–1.09)	0.084	0.014 (0.007)
Type of surgery	1.95 (1.38–2.74)	<0.001	1.35 (0.92–2.00)	0.129	0.013 (0.003)
Multifocality	2.39 (1.65–3.48)	<0.001	1.50 (1.01–2.24)	0.046	0.011 (0.006)
NAC regimens	1.14 (0.72–1.81)	0.571	1.25 (0.78–1.99)	0.351	0.005 (0.003)

The final column denotes the feature importance of the included variables in the survival model and the corresponding standard deviations (SD). The table is sorted in order of descending importance to the survival model.

*cT* clinical tumor stage, *cN* clinical node stage, *ypT* pathological tumor stage, *ypN* pathological node stage, *NAC* neoadjuvant chemotherapy.

We further assessed the independence of the model predictors by evaluating multicollinearity (Supplementary Fig. 3). The Spearman correlation matrix confirmed that all features had weak to moderate correlations (all |*r*| ≤ 0.25). The strongest, though still moderate, associations were observed between multifocality and type of surgery (*r* = 0.25) and between cN stage and multifocality (*r* = 0.18).

The results of the survival model trained on all variables were compared to those of a model only incorporating TNM staging information (cT stage and cN stage). Compared to the full model, the TNM-only model yielded a lower C-index of 0.725 (SD 0.031), at a similar calibration (0.033, SD 0.001).

Finally, the variable importances were compared to both univariate and multivariate HRs from a Cox model (Table 2) on the full cohort. In the multivariate analysis, the cN stage yielded the greatest HR of 1.73 (95% CI 1.49–2.01). This was followed by multifocality (HR 1.50; 95% CI 1.01–2.24) and cT stage (HR 1.45; 95% CI 1.15–1.81).

### Prognostic stratification into risk groups

**Fig. 1:**
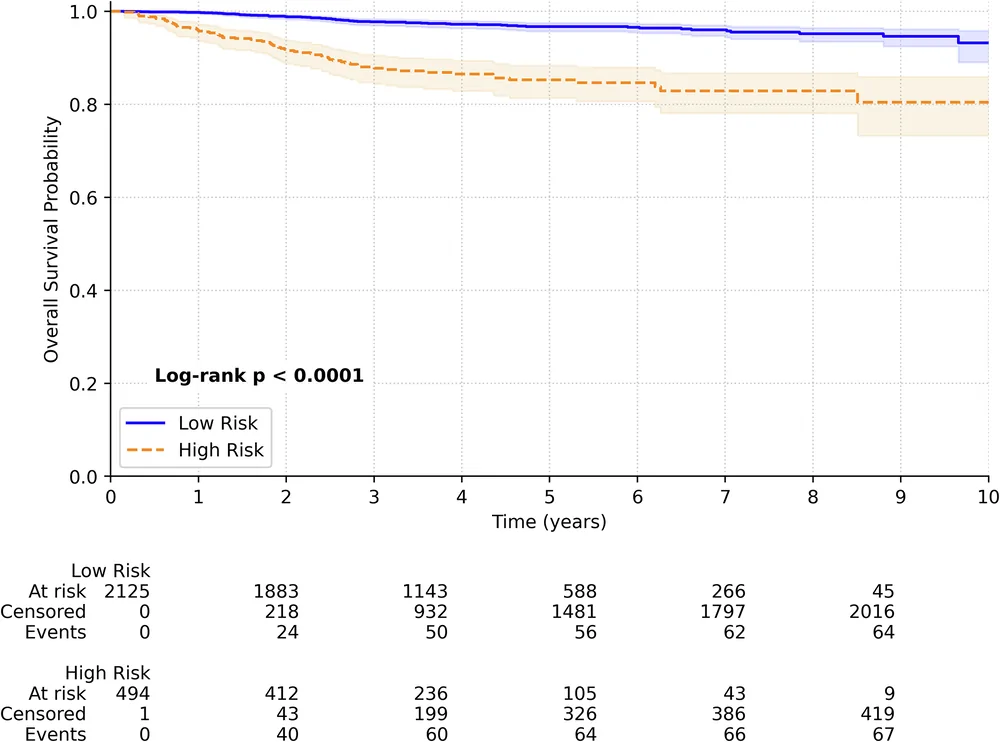
Kaplan-Meier curves for overall survival. Patients were stratified into a low-risk group (*n* = 2125) represented by a solid blue line and a high-risk group (*n* = 495) represented by a dashed orange line. The shaded areas indicate the 95% confidence intervals for each group. The *P*-value was calculated using the log-rank test.

The median time of follow-up was 4.4 years. In total, 131 (5.0%) of the patients died during the follow-up period. The threshold was set at a predicted 95% probability of 3-year OS (DCA plot displayed in Supplementary Fig. 2). Based on this threshold, two groups emerged (Fig. 1): a low-risk (*n* = 2125, 81.1%) group and a high-risk group (*n* = 495, 18.9%). This low-risk pCR group had an observed 3-year OS of 97.7%, which was 88.1% in the high-risk group (*P* < 0.001). This difference remained significant at 5 years: 96.7% survival in the low-risk group, and 85.2% in the high-risk group (*P* < 0.001).

A quantitative comparison of the two groups (Table 3) confirmed that the high-risk group was characterized by higher cT and cN stages, as well as older age (*P* < 0.001 for all). Notably, 37.6% of the patients in the high-risk group presented with cN3 stage. The high-risk group also showed significant differences in treatment and pathological characteristics compared with the low-risk group. Patients in the high-risk group more often underwent mastectomy (51.7% vs. 26.7; *P* < 0.001) and had a slightly longer median duration of NAC (20 vs. 19 weeks; *P* < 0.001). Multifocal tumors were also more frequent among high-risk patients (36.8% vs. 10.1%, *P* < 0.001). Regarding systemic therapy, anthracycline-taxane-based regimens were predominant in both groups, though taxane-based chemotherapy regimens were slightly more common in the high-risk group (*P* = 0.009).

**Table 3:** Characteristics of the low-risk and high-risk groups within the pathological complete response (pCR) cohort

Variable	Low-risk (*n* = 2125)	High-risk (*n* = 495)	*P*-value
Age	47 (40–56)	55 (45–64)	<0.001
cT stage			<0.001
I	687 (32.3)	56 (11.3)	
II	1371 (64.5)	289 (58.4)	
III	66 (3.1)	87 (17.6)	
IV	1 (0.0)	63 (12.7)	
cN stage			<0.001
0	1712 (80.6)	52 (10.5)	
I	401 (18.9)	208 (42.0)	
II	12 (0.6)	49 (9.9)	
III	0 (0.0)	186 (37.6)	
Tumor multifocality, present	214 (10.1)	182 (36.8)	<0.001
Duration of NAC [weeks]	19 (17–21)	20 (18–22)	<0.001
NAC regimens			0.009
Anthracycline and Taxane	2012 (94.7)	459 (93.3)	
Anthracycline based	72 (3.4)	15 (3.0)	
Taxane based	41 (1.9)	21 (4.2)	
Surgery Type			<0.001
Lumpectomy	1557 (73.3)	239 (48.3)	
Mastectomy	568 (26.7)	256 (51.7)	

*cT* clinical tumor stage, *cN* clinical node stage.

Using pathologically confirmed recurrences after primary surgery as a proxy for DFS, we performed an exploratory analysis of disease recurrence (Supplementary Fig. 4). This analysis showed a clear separation between the low- and high-risk groups over time, with consistently lower DFS in the high-risk group.

## Discussion

In the current study, we were able to develop a machine learning-based survival model for TNBC patients with pCR after NAC. In our prognostic model, we included baseline prognostic factors, allowing for better refinement of individual patient risk and a more direct association to OS than pCR alone.

Our survival model demonstrated robust prognostic accuracy to discriminate between high- and low-risk patients as well as strong calibration, indicating that the model’s predicted survival probabilities closely matched the actual observed survival rates in the cohort. After applying the model to the cohort, we were able to identify a group of pCR patients with a significantly higher risk of death compared to the low-risk group.

We identified cN stage as the most important predictor of OS in model. This effect is in concordance with a study by Huober et al., which found that cN was associated with a higher risk of relapse in TNBC patients who achieved pCR^8^. In another study, cN3 stage was found to be predictive of recurrence in breast cancer patients achieving pCR^16^. This suggests that pCR in the breast and lymph nodes does not always correlate with eradication of systemic disease. A high initial tumor burden may increase the probability of tumor heterogeneity and the pre-existence of chemoresistant cell clones. While NAC may successfully eliminate the primary tumor, it is possible that micrometastases formed from these resistant clones persist and lead to relapse. We expect this probability is higher in patients presenting with a higher baseline clinical stage. The importance of baseline TNM staging was confirmed by comparing the model’s performance with that of a model using only cT and cN stage. Despite providing strong prognostic information, adding the additional variables did improve the C-index and the prognostic accuracy of the model. Other established prognostic tools, such as PREDICT and CancerMath are widely used in breast cancer, but direct comparison with our patient cohort is limited, as these models do not account for the post-neoadjuvant context. In our cohort, all patients achieved pCR (ypT0N0), restricting the variability in post-treatment pathological factors.

Notably, type of surgery remained associated with survival even among patients achieving pCR. A higher risk was observed for those undergoing mastectomy compared to lumpectomy. This effect is likely twofold. Firstly, patients selected for mastectomy typically present with more advanced clinical stage, multifocal disease, or unfavorable tumor-breast size ratio, all of which reflect higher baseline risk. Secondly, differences in adjuvant treatments, such as the omission of radiotherapy after mastectomy, may contribute to this association. Finally, initial multifocality also contributed modestly to the prognostic model and differed significantly between the high- and low-risk groups. Multifocal disease was more frequent in the high-risk group.

The patients in the identified high-risk group might benefit from treatment escalation. While adjuvant therapy for the full pCR group currently does not show significant benefit^17^, this treatment may benefit the high-risk patients. New trials, such as the OptimICE-pCR (phase III) trial, will investigate the additional benefit of adjuvant pembrolizumab for TNBC patients who achieved pCR after chemo-immunotherapy^18^. The trial specifically includes stage II-III patients, a group overlapping with the high-risk pCR population identified using our model. Should OptimICE-pCR demonstrate a benefit of adjuvant immunotherapy in this group of patients, it would support the concept that baseline risk, rather than pCR status alone, should guide post-neoadjuvant treatment decisions.

Our current survival model may be expanded by incorporating more pathological biomarkers, such as tumor-infiltrating lymphocytes (TILs), which have shown prognostic significance in the TNBC subtype^19,20^. While cN showed strong prognostic significance in this study, evidence suggests that incorporation of TILs can further stratify this high-risk group^21^. However, to establish these prognostic biomarkers in real-world data, standardized reporting of TILs in routine pathology practice should be implemented to facilitate further research in refining these prognostic models. Finally, circulating tumor DNA (ctDNA) may further refine the stratification between low-risk and high-risk patients within the pCR population by combining baseline risk factors with the potential detection of residual disease^22^.

This study is based on a large, nationwide population cohort, which enhances the generalizability of our findings. The small standard deviations found in the nested cross-validation approach suggest that our models capture generalizable patterns. Our presented survival model may be implemented as a clinical calculator tool, allowing clinicians to input routinely collected variables and obtain a TNBC patient’s individualized risk score. The model provides continuous survival probabilities that may be used in clinical decision-making. In settings with limited data availability, predefined thresholds derived from this study may serve as a reference, while local calibration can be performed where sufficient outcome data are available. Alternatively, the continuous risk estimates may be interpreted without dichotomization, allowing clinicians to directly interpretate model outputs.

A limitation of this study is the reliance on OS data, due to the standard availability in our nationwide database. Other outcomes, such as DFS, may capture more subtle prognostic patterns, but are not routinely stored in the national database. The relatively young age of patients in our cohort, and the fact that they were in physical condition to receive NAC makes death from other causes within three years unlikely. The OS endpoint, however, is affected by subsequent post-NAC treatments following potential relapse, and the exclusion of patients who received non-standardized treatment may further introduce selection bias. We attempted to approximate DFS, defined as any pathologically confirmed recurrence of disease after primary tumor resection. This approach confirmed the validity of the identified risk-groups. However, as not all recurrences result in pathological confirmation, this approach does not allow for complete and reliable ascertainment of recurrence. The exploratory analysis should therefore be interpreted with caution. Furthermore, due to unavailability of the tumor diameter, we used cT stage as measure for initial tumor burden. Including this diameter instead of cT may make the risk predictions more detailed. Finally, this study used data from a single country. Although based on standardized variables like TNM staging, its performance may be influenced by international differences in population characteristics, data registration practices, and specific neoadjuvant treatment protocols. Validation in external, international cohorts is therefore a required next step to ensure its generalizability before wider clinical implementation.

In conclusion, this study establishes machine learning-based risk stratification models for TNBC patients with pCR following NAC, moving beyond the traditional binary pCR to provide individualized survival prediction. Our survival model, utilizing only routinely collected clinical variables, is a candidate tool for providing guidance for personalized TNBC treatment decisions after pCR. Future studies should investigate the actual benefit of adjuvant treatment in these patients.

## Methods

### Patient cohort

A nationwide retrospective dataset of TNBC patients diagnosed between 2013 and 2021 was compiled by the Netherlands Comprehensive Cancer Organization (IKNL) using the Netherlands Cancer Registry (NCR). This dataset, containing 14,920 TNBC patients, was linked to corresponding pathology reports in the Dutch Nationwide Pathology Databank (Palga)^23^. The most recent update on overall survival (OS) was performed in February 2024. Patients were considered TNBC when ER and PR expression was <10% and the HER2 immunohistochemistry was 0, 1+, or 2+ with a negative reflex test^24,25^. We used the most common definition for pCR, also defined in the AJCC TNM staging: no remaining invasive tumor in the breast and lymphnodes (ypT0N0), but allowing for in-situ disease (ypTis)^24,25^.

**Fig. 2:**
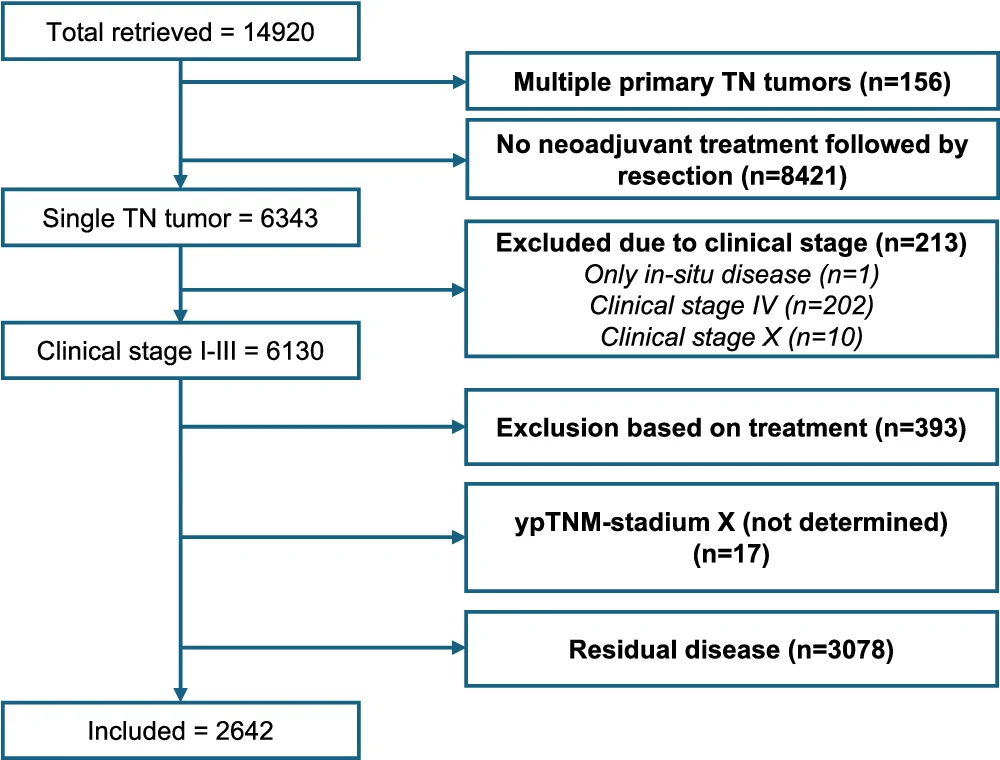
Patient selection flowchart for the study cohort. Schematic representation of the inclusion and exclusion criteria applied to the nationwide Dutch dataset. A total of 14,920 triple-negative breast cancer cases were initially retrieved from the Netherlands Cancer Registry (IKNL) and linked to the Dutch Nationwide Pathology Databank (Palga). The final analysis focused on 2642 patients who achieved a pathological complete response (pCR).

As shown in the flowchart (Fig. 2), patients were excluded if they had a history of breast cancer or a synchronous breast tumor (*n* = 156), or if the clinical stage of the tumor was IV (*n* = 202), unknown (*n* = 10), or only in-situ disease (*n* = 1). Further exclusions involved patients who did not receive NAC (*n* = 8421) and those for whom the post-NAC pathological stage (ypTNM) was not determined (*n* = 17). The treatment regimens of the remaining cases were screened. Patients were included if the NAC regimen was anthracycline- and/or taxane-based. To increase the power of prediction for standard-treated cases, patients were excluded if: (i) a patient received postoperative chemotherapy, (ii) the NAC regimen was monotherapy with a taxane without cyclophosphamide or a platinum-based agent, or (iii) patients received experimental treatment within a clinical trial (totaling *n* = 393). Finally, we excluded all patients with residual disease after NAC (*n* = 3078), leaving 2642 patients who achieved a pCR after NAC.

This study was performed in accordance with the Declaration of Helsinki. The study protocol and data request were reviewed and approved by the NABON-BOOG committee (on behalf of IKNL; request no. K24.011) and the Scientific Council and Privacy Committee of Palga (request no. lzv2024-27). According to the Dutch Medical Research Involving Human Subjects Act (WMO), this study was exempt from formal review by a Medical Research Ethics Committee (MREC) as it involved the retrospective analysis of existing, pseudonymized data. Consequently, the requirement for individual informed consent was waived by the aforementioned committees. Data were collected by IKNL and Palga based on an opt-out system in accordance with Dutch legislation (WMO and GDPR); patients are informed of their right to object to the use of their data for scientific research, and only data from patients who did not object were included.

### Survival model development

We included age at time of the diagnostic biopsy, pre-NAC clinical tumor size (cT), and clinical lymph node involvement (cN) according to the TNM classification^26^, NAC regimens, duration of the treatment (in weeks), tumor multifocality, pathological tumor type, and grading from the NKR dataset and pathology reports.

The dataset contained missing values (numbers shown in Table 1). For the survival analysis, we excluded the patients with missing values for either cT stage (*n* = 2), cN stage (*n* = 15), duration of NAC (*n* = 3), or surgery type (*n* = 2). As these 22 patients collectively represented only 0.8% of the cohort, this complete case exclusion was performed rather than data imputation. For further analysis, only data of the remaining 2620 patients were used.

**Fig. 3:**
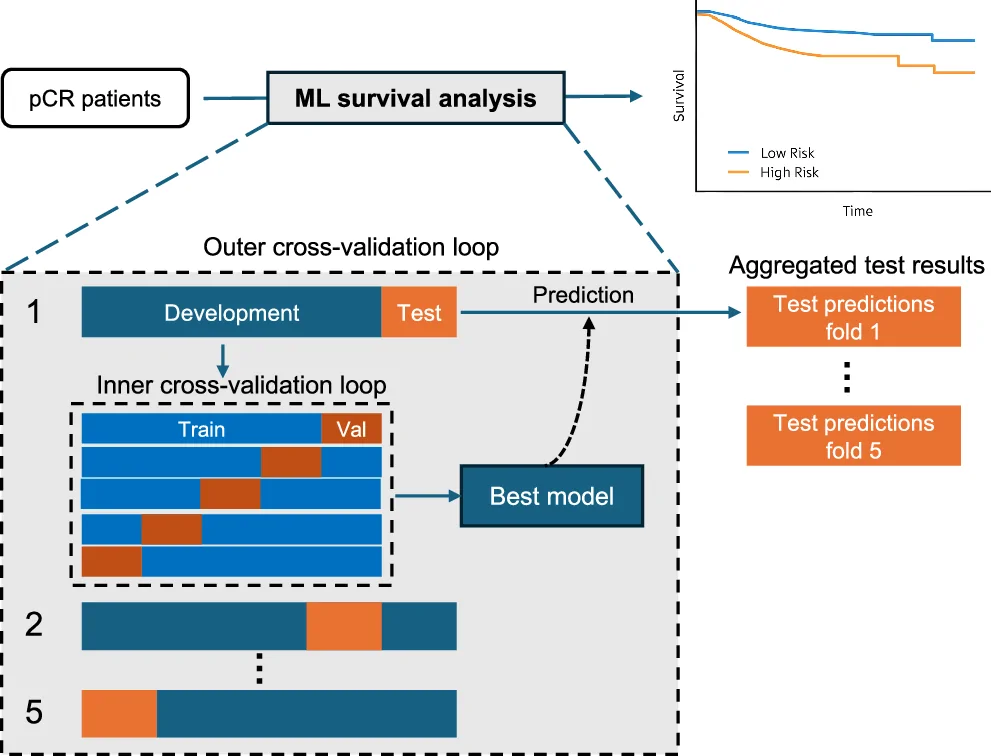
Overview of the methodology in this study. The survival model was trained using a nested cross-validation framework. First, the cohort was randomly split into 80% development and 20% hold-out test data. Within the development set, an inner cross-validation loop was applied. The data were repeatedly partitioned into training and validation subsets to optimize model performance and select the best-performing survival model. This best model was then retrained on the full development set and used to generate predictions on the hold-out test set. This process was repeated across five outer folds to ensure robustness and reduce overfitting, resulting in five sets of test predictions. The aggregated predictions were subsequently used for survival stratification into high- and low-risk groups.

The primary endpoint was OS, defined as time from the date of breast surgery to the date of death from any cause. To ensure the model was robust and generalizable to new patients, we employed a validation technique known as nested 5-fold cross-validation^27^. As shown in Fig. 3, this method repeatedly splits the data into a training set, to build the model, and a separate test set, to evaluate its performance on unseen data. These training and test sets enable an unbiased estimate of performance. Within this process, we tested several machine learning survival analysis algorithms, including Cox proportional hazard, survival trees and random survival forests^28^, gradient boosting survival analysis^29^, and Cox proportional hazards models with elastic net regularization (CoxNetSurvivalAnalysis)^30^. In case of the Cox models, the baseline survival functions were estimated using Breslow’s estimator, such that the output of the models were absolute survival probabilities.

### Evaluation of survival model performance

The survival models were evaluated using three metrics: (i) the Harrell’s Concordance Index (C-index), which measures how well the model ranks patients according to their survival time, where a value closer to one indicates better discrimination, (ii) the time-dependent Area Under the Receiver Operating Characteristic curve (AUC) at clinically relevant time points (1, 3, and 5 years), and (iii) the Integrated Brier Score (IBS), which evaluates the calibration (i.e. the overall accuracy of the survival predictions over time), where a lower score indicates better model calibration^31^. The IBS was used as primary metric to select the best model in the cross-validation.

To create the final risk groups, an unbiased risk prediction for each patient was first generated by aggregating the predictions from the hold-out test set of each outer fold. A single, optimal stratification threshold was then derived by analyzing the model’s performance exclusively within the development data splits to avoid data leakage. We empirically evaluated thresholds based on predicted 3-year and 5-year OS probabilities, assessing their ability to form clinically meaningful and well-separated prognostic groups. Importantly, threshold selection was guided by a predefined clinical criterion: the low-risk group was required to have an observed 3-year OS of at least 97%, reflecting a population with a very low risk of early mortality. We subsequently used decision curve analysis (DCA) to confirm a positive net benefit for model-based stratification at the identified threshold.

Finally, we employed permutation variable importance to identify the most influential predictors in the model^32^. This technique measures how much a model’s performance decreases when the information from a single variable is randomly shuffled, with larger drops indicating greater importance.

We performed an exploratory analysis to approximate disease-free survival (DFS), defined as any pathologically confirmed recurrence of disease after primary tumor resection. However, as not all recurrences result in pathological confirmation, this approach provides an incomplete proxy for true recurrence and was therefore considered exploratory.

### Statistical analysis

General characteristics of the cohort were compared using the independent Student's *t*-test or the Mann–Whitney *U* test, where appropriate. To assess for potential multicollinearity between ordinal and continuous model features, a Spearman’s rank correlation matrix was calculated and visualized. The survival differences between the low-risk and the high-risk groups were assessed using Kaplan–Meier (KM) estimators and statistically compared using the log-rank test. All analyses were performed using Python (version 3.12) with scikit-survival (version 0.24) and lifelines (version 0.30) packages^27,33^. A two-sided *p*-value below 0.05 was considered statistically significant. The mean and standard deviation (SD) of all performance metrics (C-index, IBS, and AUC) were calculated across the five held-out test folds of the outer cross-validation loop. Specifically, for each outer fold, a single performance metric was computed on the corresponding held-out test set, resulting in five values per metric, from which the mean and SD were derived.

## Supplementary information


Supplementary Information


## Data Availability

The datasets analyzed during the current study are not publicly available due to legal and contractual data protection restrictions imposed by IKNL and Palga. These data were used under license for the current study and cannot be shared publicly.

## Code availability

The underlying code for this study is not publicly available but may be made available to qualified researchers on reasonable request from the corresponding author.
