# Supplementary material for: Machine learning-based identification of high-risk triple-negative breast cancer patients despite pathological complete response after neoadjuvant chemotherapy
Source: NPJ Breast Cancer. 2026 Jun 2;12:114. doi: 10.1038/s41523-026-00979-0 (PMC13526796; doi:10.1038/s41523-026-00979-0)
Supplement: Supplementary file 1 [file 41523_2026_979_MOESM1_ESM.pdf]

Machine learning-based identification of high-risk triple-negative breast cancer patients despite pathological complete response after neoadjuvant chemotherapy

Koen Kwakkenbos, Nadine S van den Ende, Bernadette AM Heemskerk-Gerritsen, Jifke F Veenland, Agnes Jager, Carolien HM van Deurzen

Supplementary data file, containing:

- **Supplementary Figure 1**
- **Supplementary Figure 2**
- **Supplementary Figure 3**
- **Supplementary Figure 4**

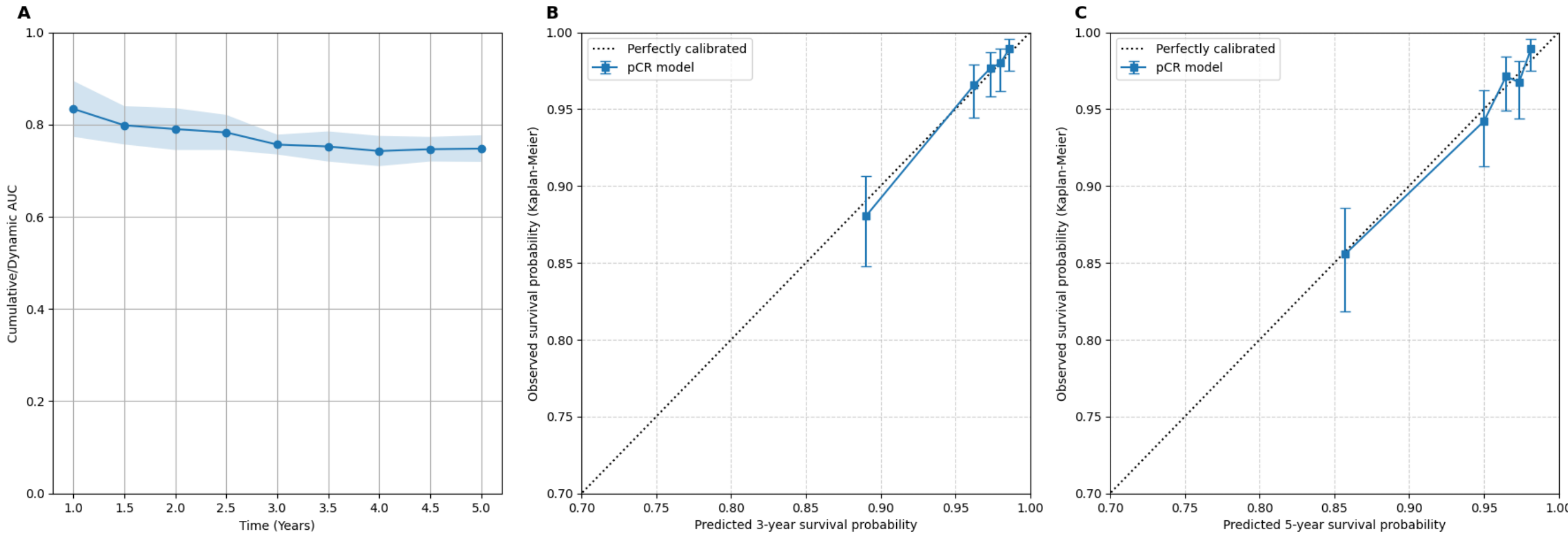

Supplementary Figure 1: Performance and calibration of the survival model. a) Dynamic area under the curve (AUC) for the survival model from 1 to 5 years post-surgery. The solid blue line represents the mean AUC, and the shaded light-blue area indicates the 95% confidence interval. b) Calibration plot for 3-year overall survival prediction. c) Calibration plot for 5-year overall survival prediction. In panels b and c, the dashed black line represents perfect calibration, and the blue squares with error bars represent the observed survival (Kaplan-Meier) versus the predicted survival probability for the pCR model. Abbreviations: AUC, area under the curve; pCR, pathological complete response.

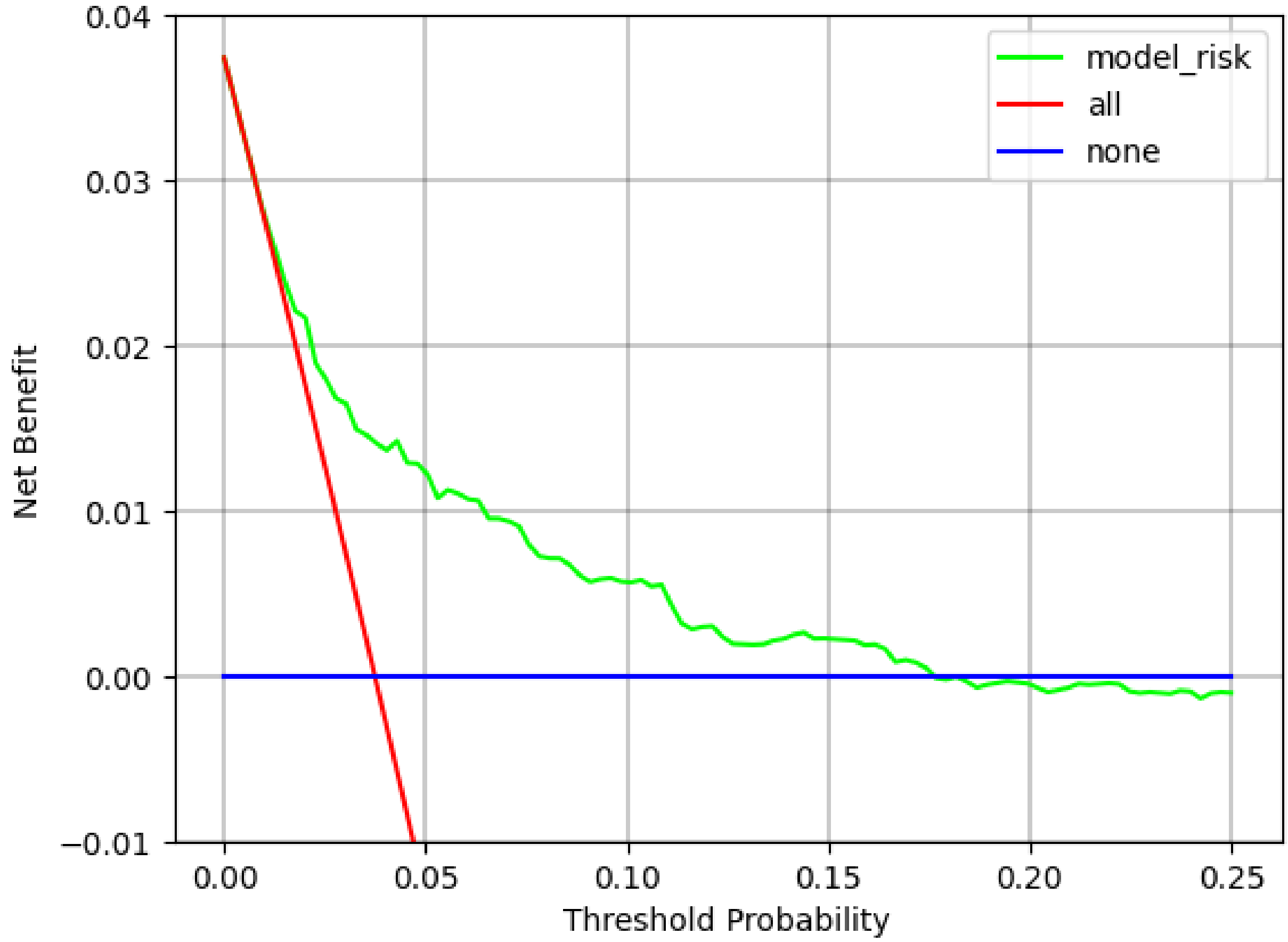

Supplementary Figure 2: Decision curve analysis plot for determination of the threshold. The plot illustrates the net benefit of using the model-based risk stratification compared to the "all" (treating everyone as high risk) and "none" (treating no one as high risk) strategies. The solid green line represents the model-based risk, the solid red line represents the "all" strategy, and the solid blue line represents the "none" strategy. The x-axis values represent one minus the chosen probability threshold.

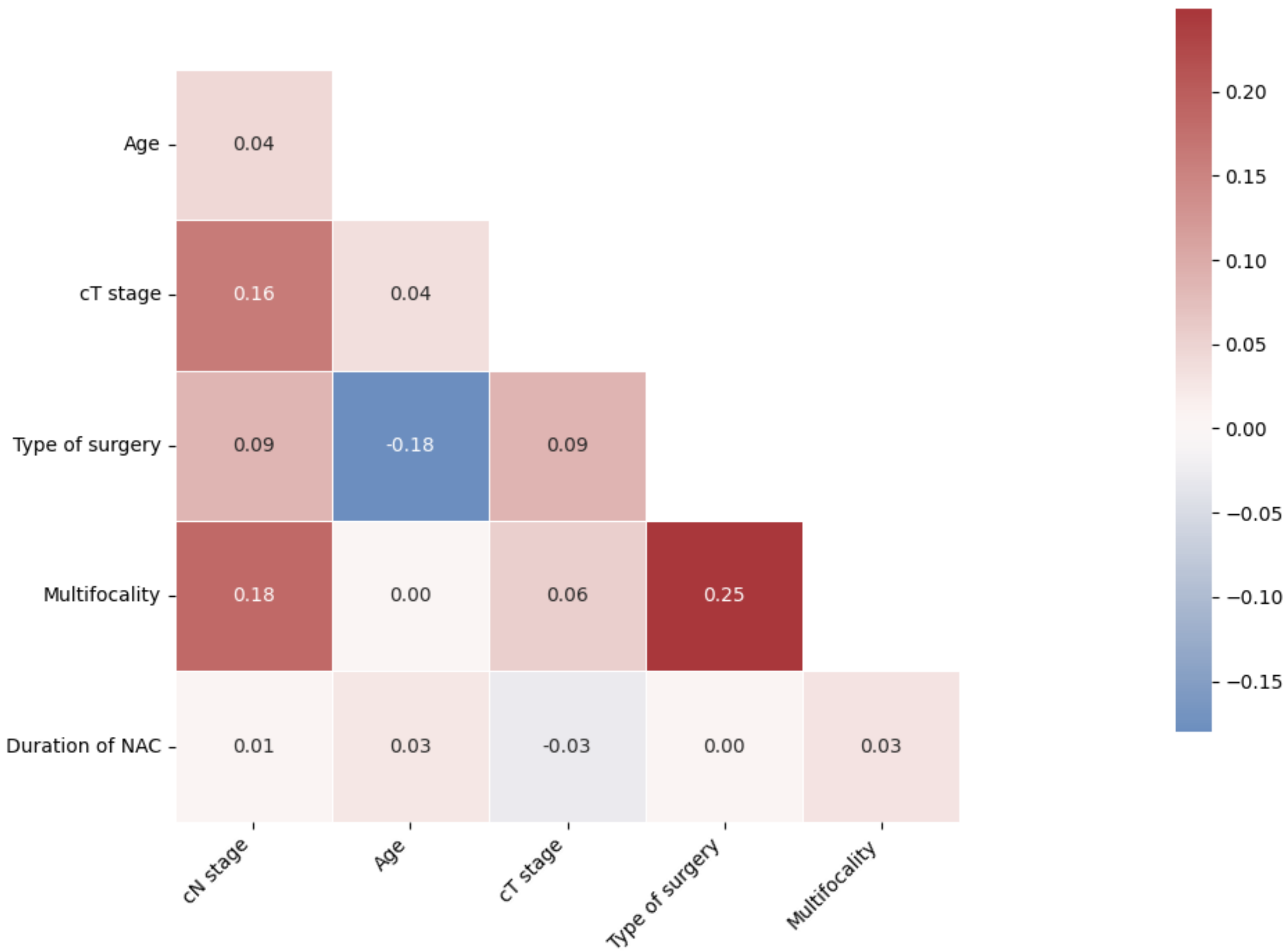

Supplementary Figure 3: Spearman correlation matrix of clinical and pathological features. Heatmap showing the correlation coefficients between variables used in the survival model. Positive correlations are shown in shades of red, and negative correlations are shown in shades of blue. The numerical values within each cell indicate the Spearman's rank correlation coefficient. cN: clinical node stage; cT: clinical tumor stage; NAC: neoadjuvant chemotherapy.

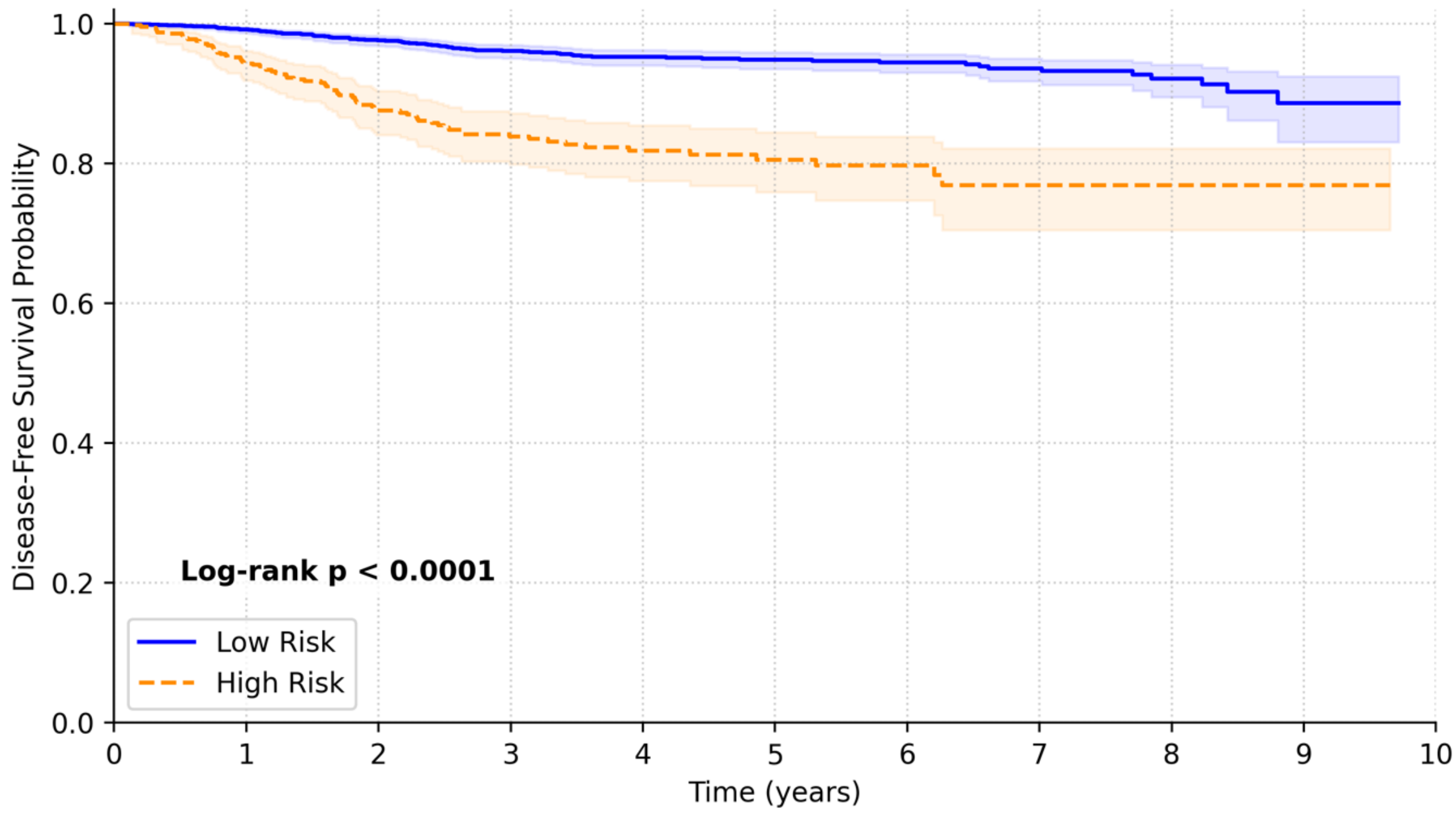

**Low Risk**						
At risk	2124	1494	827	406	140	0
Censored	1	587	1224	1640	1900	2037
Events	0	44	74	79	85	88
**High Risk**						
At risk	494	312	175	64	23	0
Censored	1	129	249	357	396	419
Events	0	54	71	74	76	76

Supplementary Figure 4: Exploratory analysis using pathologically confirmed recurrences after primary surgery as a proxy for disease-free survival. Kaplan-Meier curves show the disease-free survival (DFS) for patients in the low-risk group (solid blue line) and high-risk group (dashed orange line). Risk groups were assigned based on the primary survival model. Shaded areas represent the 95% confidence intervals. The P-value was calculated using a log-rank test. Recurrence events are likely underrepresented as only those with pathological confirmation were included.
